# Relevance of Coding Variation in *FLG* And *DOCK8* in Finnish Pediatric Patients with Early-Onset Moderate-To-Severe Atopic Dermatitis

**DOI:** 10.1016/j.xjidi.2023.100203

**Published:** 2023-04-04

**Authors:** Miia Perälä, Meri Kaustio, Alexander Salava, Eveliina Jakkula, Anna S. Pelkonen, Janna Saarela, Anita Remitz, Mika J. Mäkelä

**Affiliations:** 1 Skin and Allergy Hospital, Helsinki University Hospital, University of Helsinki, Helsinki, Finland; 2 Institute for Molecular Medicine Finland (FIMM), University of Helsinki, Helsinki, Finland; 3 Department of Clinical Genetics, Helsinki University Hospital, University of Helsinki, Helsinki, Finland; 4 Centre for Molecular Medicine Norway, University of Oslo, Oslo, Norway

## Abstract

Early-onset, persistent atopic dermatitis (AD) is proposed as a distinct subgroup that may have specific genotypic features. *FLG* gene loss-of-function variants are the best known genetic factors contributing to epidermal barrier impairment and eczema severity. In a cohort of 140 Finnish children with early-onset moderate-to-severe AD, we investigated the effect of coding variation in *FLG* and 13 other genes with epidermal barrier or immune function through the use of targeted amplicon sequencing and genotyping. A *FLG* loss-of-function variant (Arg501Ter, Ser761fs, Arg2447Ter, or Ser3247Ter) was identified in 20 of 140 patients showing higher transepidermal water loss values than patients without these variants. Total *FLG* loss-of-function variant frequency (7.14%) was significantly higher than in the general Finnish population (2.34%). When tested separately, only Arg2447Ter showed a significant association with AD (*P* = 0.003104). In addition, a modest association with moderate-to-severe pediatric AD was seen for rs12730241 and rs6587667 (*FLG2*:Gly137Glu). Loss-of-function variants, previously reported pathogenic variants, or statistically significant enrichment of nonsynonymous coding region variants were not found in the 13 candidate genes studied by amplicon sequencing. However, higher IgE and eosinophil counts were found in carriers of potentially pathogenic *DOCK8* missense variants, suggesting that the role of *DOCK8* variation in AD should be further investigated in larger cohorts.

## Introduction

Studies have suggested that patients with atopic dermatitis (AD) can be divided into subgroups not only on the basis of clinical phenotypes but also on the basis of biomarker and genotype status (Bieber et al., 2017). One such subgroup is early**-**onset AD with *FLG* loss-of-function (LoF) variants, increased asthma risk, high IgE levels, and parental AD history (Amat et al., 2018; Drislane and Irvine, 2020). Monomeric FLG protein is produced by cleavage from a large pro-FLG precursor containing 10−12 FLG repeats and is encoded by the *FLG* gene located in the epidermal differentiation complex (review by Sandilands et al. [2009]). FLG interacts with keratins in the skin, contributing to the formation of the uppermost layer of the epidermis (stratum corneum) and the natural moisturizing factor (Sandilands et al., 2009). The insufficiency of *FLG* results in impairment of the epidermal barrier and enhanced transepidermal water loss (TEWL), making the skin more permeable and vulnerable to diverse irritants, allergens, and pathogens (Kezic et al., 2008; Sandilands et al., 2009; Smith et al., 2006). Consequently, *FLG* LoF variants predispose to AD and ichthyosis vulgaris and affect eczema severity (Palmer et al., 2006, Smith et al., 2006) (review by Liang et al. [2016]).

*FLG2* is part of the same gene family and similar in protein structure to FLG (Wu et al., 2009). Similar to FLG, FLG2 is found in the human epidermis where it is needed for proper cornification (Pendaries et al., 2015). Decreased *FLG2* expression has been detected in human skin diseases (Makino et al., 2014), and homozygosity for *FLG2* LoF variants causes the rare genodermatosis peeling skin syndrome (Alfares et al., 2017; Bolling et al., 2018; Mohamad et al., 2018). However, in addition to being linked to more persistent AD in one study on African Americans, there is not much information on the effect of *FLG2* variation on AD (Margolis et al., 2014).

In addition to *FLG* variants, associations with higher risk or severity of AD have been found for variants in many other genes affecting epidermal barrier integrity or the immune response (Liang et al., 2016; Martin et al., 2020). To study the genetics behind moderate-to-severe pediatric AD in Finland, we investigated the relevance of sequence variation in *FLG* and *FLG2* as well as in 12 other genes with a previous connection to AD pathogenesis in a cohort of Finnish pediatric patients with early-onset AD.

## Results

**Figure 1:**
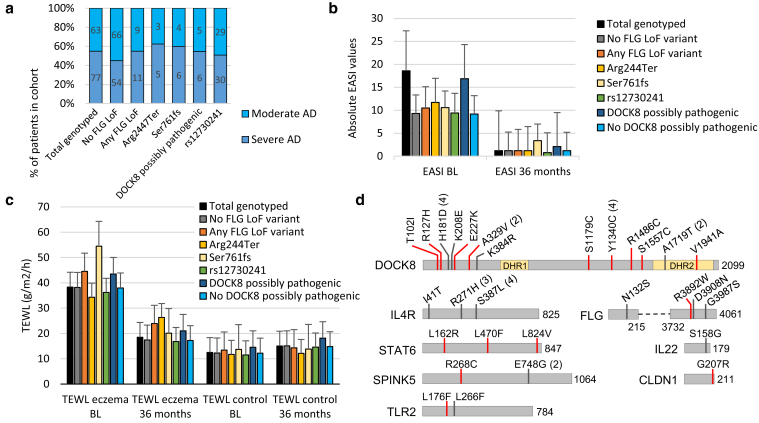
**Distribution of AD severity, EASI, and TEWL in patients with and without *FLG* and *DOCK8* variants and schematic overview of candidate genes’ protein sequences.** (**a**) Severity of AD in relation to *FLG* and *DOCK8* variant status. (**b**) EASI values in relation to *FLG* and *DOCK8* variant status. (**c**) TEWL (g/m2/h) at eczema and control sites, at BL, and at 36 months. (**d**) Schematic representation of candidate genes’ protein sequences with positions of rare exonic variants detected by amplicon sequencing marked with vertical lines. Red lines = scaled CADD score > 15 (higher likelihood of pathogenicity), and gray lines = scaled CADD score < 15 (lower likelihood of pathogenicity). If variant was detected in multiple individuals, the number of carriers is marked in brackets. Dashed line = region in *FLG* not targeted by amplicons. AD, atopic dermatitis; BL, baseline; CADD, Combined Annotation Dependent Depletion; EASI, Eczema Area and Severity Index; LoF, loss of function; pos.pathogenic, possibly pathogenic; STAT6, signal transducer and activator of transcription 6; TEWL, transepidermal water loss; TLR2, toll-like receptor 2.

We studied 140 children with moderate-to-severe disease at the Skin and Allergy Hospital in Helsinki (Helsinki, Finland) as part of a 3-year randomized open-label follow-up study (Perälä et al., 2023). The baseline demographics of the study population are shown in Table 1 and Figure 1a. To determine the significance of *FLG* variations for Finnish pediatric AD, we genotyped selected single-nucleotide variations (SNVs) in the *FLG* (n = 8) and *FLG2* (n = 6) genes and tested their association with AD using Fisher’s exact test (Løset et al., 2019) and Benjamini−Hochberg false discovery rate correction for multiple testing (Benjamini and Hochberg, 1995). Variants detected in the patient cohort included the four most prevalent European *FLG* LoF variants Arg501Ter, Ser761fs, Arg2447Ter and Ser3247Ter, and rs12730241 (G>A) and three *FLG2* variants (Ser2377Ter, Cys298Ser, and Gly137Glu). Other genotyped loci included Gln1754Ter, Ser1020Ter, and Val603Met for *FLG* and Thr1314fs, Cys298Arg, and Leu168Phe for *FLG2*, but they were monomorphic in patients. Allele frequencies, carrier numbers, and association results are shown in Table 2.

**Table 1:** Baseline Demographics

Variables	Number
Patients	140
Male, n (%)	73 (52)
Age (y), median (Q1−Q3)	1.7 (1.3−2.3)
Severity of AD^1^: moderate/severe, n (%)	77 (55)/63 (45)
Family history of atopy^2^, n (%)	120 (90)
Parent(s) smoking, n (%)	39 (30)
Pets, n (%)	31 (24)
Age (mo) at solid food introduction, median (Q1−Q3)	5.0 (4.0−6.0)

Abbreviations: AD, atopic dermatitis; Q1, quartile 1; Q2, quartile 3.

Results are presented as medians with 25th−75th percentiles (Q1−Q3).

1 According to the Rajka−Langeland criteria.

2 AD, allergic rhinitis, or asthma.

**Table 2:** Association Results for Genotyped *FLG* and *FLG2* Variants

Gene	SNV (rsNumber)	Major (Reference) Allele	Minor Allele	Protein Change	*P* (unadjusted)	*P* (FDR)	OR	Study Cohort (Pediatric AD)				Control Cohort (H2000 GenMets)			
								N_hom	N_het	N_hom_ref	MAF (%)	N_hom	N_het	N_hom_ref	MAF (%)
*FLG*	rs138726443	G	A	Arg2447Ter	0.0002217	**0.003104**	5.8	0	8	131	2.88	0	17	1645	0.51
*FLG*	rs12730241	G	A	NA (12-repeat marker)	0.004009	**0.02807**	1.5	10	49	81	24.64	45	490	1124	17.48
*FLG2*	rs6587667	C	T	Gly137Glu	0.008428	**0.03933**	3.6	0	6	134	2.14	0	20	1613	0.61
*FLG*	rs558269137	ACTG	-	Ser761fs	0.01939	0.06788	2.4	0	10	129	3.60	2	48	1614	1.56
*FLG*	rs150597413	G	T	Ser3247Ter	0.1119	0.3133	6.2	0	1	133	0.37	0	2	1657	0.06
*FLG*	rs61816761	G	A	Arg501Ter	0.1531	0.3571	4.0	0	1	137	0.36	0	3	1652	0.09
*FLG2*	rs12568784	G	T	Ser2377Ter	0.4517	0.6085	1.1	4	36	100	15.71	40	393	1226	14.26
*FLG2*	rs2282302	C	G	Cys298Ser	0.4672	0.6085	1.1	6	51	83	22.50	66	554	1038	20.69
*FLG*	rs769696694	G	A	Gln1754Ter	0.5388	0.6085	0	0	0	140	0	0	1	1663	0.03
*FLG2*	rs567184084	-	TA	Thr1314fs	0.5388	0.6085	0	0	0	140	0	0	1	1663	0.03
*FLG2*	rs145678751	A	G	Cys298Arg	0.5747	0.6085	0	0	0	140	0	0	2	1660	0.06
*FLG2*	rs61749580	T	A	Leu168Phe	0.607	0.6085	0	0	0	139	0	0	3	1661	0.09
*FLG*	rs200360684	G	C	Ser1020Ter	0.6076	0.6085	0	0	0	140	0	0	3	1661	0.09
*FLG*	rs137995883	C	T	Val603Met	0.6085	0.6085	0	0	0	140	0	0	3	1645	0.09
*FLG*	any LoF				2.72E-05		3.2	20	0	120	7.14	2 hom 1 comp het	72	1664	2.34

Abbreviations: AD, atopic dermatitis; FDR, false discovery rate; H2000, Health 2000; LoF, loss of function; MAF, minor allele frequency; Ref, reference.

N_hom denotes the number of homozygotes for minor allele, N_het denotes the number of heterozygotes, and N_hom_ref denotes the number of homozygotes for the reference allele.

Protein change refers to the following RefSeq transcripts and proteins: FLG: NM_002016.1, NP_002007.1; FLG2: NM_001014342.2, NP_001014364.1. Significant *P*-values are bolded.

A total of 20 of the 140 patients were heterozygous for an *FLG* LoF variant, which translated into a significantly higher combined *FLG* LoF-variant frequency of 7.14% in patients compared with 2.34% in controls (*P* = 2.72E-05, OR = 3.2). Although the small cohort size and low variant frequencies limited our ability to detect statistically significant associations for SNVs, the Arg2447Ter variant (n = 8) showed a significant association with AD (*P* = 0.003104, OR = 5.8). A modest association was also detected for rs12730241 (*P* = 0.028, OR = 1.5) and rs6587667 (*P* = 0.039, OR = 3.6). The rs6587667 variant co-occurred with the rs12730241 variant in 19 of 20 controls and in six of six patients, indicating that the two variants are in linkage disequilibrium and not independent. Hence, the contributions of these SNPs to the association signal as well as their causality should be evaluated in more detail by further studies on bigger cohorts.

**Figure 2:**
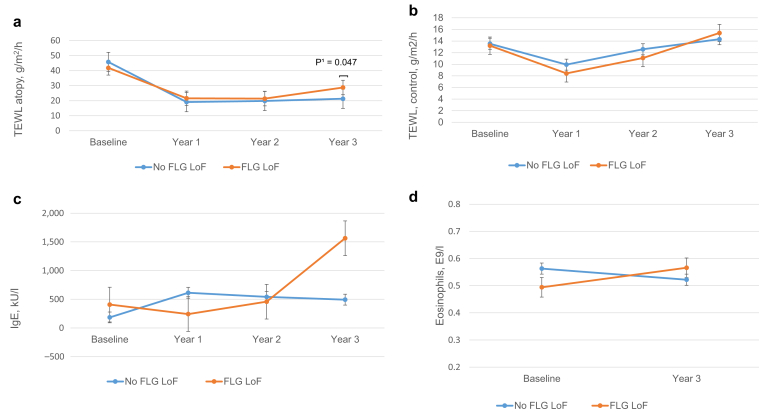
**Change in eczema treatment parameters (estimated marginal means) during the study in *FLG* LoF variant carriers and noncarriers.** (**a**) TEWL (g/m2/h) at the eczema site. (**b**) TEWL (g/m2/h) at the control site. (**c**) IgE (kU/l). (**d**) Eosinophils (E9/l). The superscript number (^1^) denotes repeated measures ANOVA with Bonferroni correction. LoF, loss of function; TEWL, transepidermal water loss.

*FLG* LoF-variant carrier status was not linked to disease severity, whereas TEWL at previous eczema site was significantly higher at 36 months in LoF carriers than in noncarriers (Table 3 and Figure 1a−c) (*P* = 0.029). In addition, carriers of Ser761fs had significantly higher eczema TEWL at baseline (*P* = 0.021). Changes in eczema treatment parameters (estimated marginal means) during the study in *FLG* LoF-variant carriers and noncarriers are shown in Figure 2a−d. Occurrence of rs12730241-A allele correlated with significantly lower TEWL at the study end (*P* = 0.036). There were no differences in clinical parameters between rs12730241-A allele homozygotes (n = 10) and heterozygotes (n = 49).

**Table 3:** Clinical Data in Relation to *FLG* Status

*FLG* Variant	n		BSA	EASI	IGA	TEWL Control	TEWL Eczema	*P*-Value
	Het/Hom		%	Median (Q1-Q3)		Median (Q1−Q3)	Median (Q1−Q3)	
Total genotyped	140	BL mo 36	18.6 (1.1−38.8) 1.2 (0−4.4)	9.3 (6.5−16.4) 1.2 (0−3)	3 (3−4) 1 (0−2)	12.45 (9.3−15.8) 15.0 (11.1−17.4)	38.3 (27.9−49.3) 18.5 (13.8−27.9)	
No *FLG* LoF variant	120	BL mo 36	18 (10.6−37.6) 1.2 (0−4.1)	9.3 (6.4−16.2) 1.2 (0−2.5)	3 (3−4) 1 (0−2)	12.3 (8.6−16.1) 15.1 (10.1−17.6)	38.2 (28.3−44.9) 17.4 (12.9−26.7)	
*Any FLG* LoF variant	20/0	BL mo 36	24.4 (11.9−49) 2.5 (0.2−9.1)	10.5 (6.6−19.7) 1.2 (0.3−4.7)	3.5 (3−4) 1.5 (0.8−3)	13.4 (10.1−15) 14.3 (11.6−17)	44.5 (24.9−55.5) **23.9 (17−34.5)**^1^	**0.029**
*FLG*:Arg2447Ter	8/0	BL mo 36	28 (18.6−51.5) 1.9 (0.2−3.4)	11.7 (7.5−24.7) 1.2 (0.6−2.8)	4 (3.3−4) 1 (1−2)	11.7 (8.6−12.6) 12.1 (11.2−15.2)	34.3 (24.9−44.5) 26.3 (17.4−31.2)	
*FLG*:Ser761fs	10/0	BL mo 36	18.4 (7.5−49) 4 (0.1−15.7)	10.6 (5.9−19.6) 3.4 (0.2−6.2)	3 (3−4) 2 (0.5−3)	13.7 (10.4−18.3) 13.8 (12.5−17.4)	**54.5 (44.2−72.8)**^1^ 20.1 (15.9−38.7)	**0.021**
*FLG*:Arg501Ter	1/0	BL mo 36	24 (24−24) 7.5 (7.5−7.5)	6.6 (6.6−6.6) 3.6 (3.6−3.6)	3 (3−3) 3 (3−3)	17.3 (17.3−17.3) 16.9 (16.9−16.9)	25.2 (25.2−25.2) 33.4 (33.4−33.4)	
*FLG*:Ser3247Ter	1/0	BL mo 36	40 (40−40) 0 (0-0)	8.6 (8.6−8.6) 0 (0−0)	3 (3−3) 0 (0−0)	13.8 (13.8−13.8) 16.2 (16.2−16.2)	15 (15−15) 15.2 (15.2−15.2)	
rs12730241	49/10	BL mo 36	18.3 (9.9−40.3) 0.9 (0−2.9)	9.4 (6.1−16.8) 0.8 (0−2.2)	3 (3−4) 1 (0−2)	11.5 (7.6−17) 14.6 (9.9−17.8)	36.2 (24.5−44.3) **16.8 (11.4−25)**^1^	**0.036**

Abbreviations: BL, baseline; BSA, body surface area; EASI, Eczema Area and Severity Index; Het, heterozygote; Hom, homozygote; IGA, Investigator’s Global Assessment; LoF, loss of function; Q1, quartile 1; Q2, quartile 2; TEWL, transepidermal water loss.

IGA: a five-point tool for evaluating eczema severity: 0 = clear, 1 = almost clear, 2 = mild, 3 = moderate, and 4 = severe.

1 Results (in bold) refer to statistical significance compared with noncarriers.

We also conducted an exploratory candidate-gene study for possible, to our knowledge, previously unreported monogenic-like causes of moderate-to-severe pediatric AD by amplicon sequencing protein-coding regions of the following genes: *FLG*, *CLDN1*, *DOCK8*, *IL13*, *IL17A*, *IL22*, *IL31*, *IL33*, *IL4*, *IL4R*, *SPINK5*, signal transducer and activator of transcription 6 gene *STAT6*, and toll-like receptor 2 gene *TLR2.* Gene selection for the amplicon panel was based on the genes matching one or more of the following criteria: (i) cause for monogenic immunodeficiency with AD-like features (eczema, asthma, allergy); (ii) previously reported association with AD, allergy, or asthma; or (iii) known involvement in AD pathogenesis (Table 4). Unfortunately, owing to technical challenges in designing primers for highly homologous regions, we failed to cover all *FLG* gene coding regions. Hence, analysis of the *FLG* gene was limited to variants in nonhomologous regions at the ends of the gene and to the variants covered by genotyping.

**Table 4:** Analyzed Genes

Gene	Protein	Possible Previous Association	References	
*CLDN1*	Claudin-1		Skin barrier integrity, high serum IgE levels, and eosinophil count When decreased, early-onset AD	Asad et al. (2016), Bergmann et al. (2020), and De Benedetto et al. (2011).
*DOCK8*	Dedicator of cytokinesis protein 8		Hyper IgE syndrome, food allergies, eczema	Akdis et al. (2020), Aydin et al. (2015), and Engelhardt et al. (2015).
*FLG*^1^^˒^^2^	FLG		Ichthyosis vulgaris, eczema, eczema severity, allergies, asthma	Akdis et al. (2020), Chan et al. (2018), Martin et al. (2020), and Sandilands et al. (2009).
*FLG2*^1^	FLG2		Eczema, eczema severity	Margolis et al., (2014) and Yang et al. (2020).
*IL4*	IL-4		Asthma, eczema	Akdis et al. (2020), Furue (2020), Løset et al. (2019), Martin et al. (2020), and Yang et al. (2020).
*IL4R*	IL-4Ra		Eczema	Akdis et al. (2020), Furue (2020), Løset et al. (2019), Martin et al. (2020), and Yang et al. (2020).
*IL13*	IL-13		High serum IgE levels, food allergies, eczema	Akdis et al. (2020), Furue (2020), Løset et al. (2019), Martin et al. (2020), and Yang et al. (2020).
*IL17A*	IL-17A		Asthma, eczema severity	(Brunner et al., 2018), Esaki et al. (2016), and Furue (2020).
*IL22*	IL-22		Skin barrier damage, epidermis hyperplasia, proinflammatory mediator	Brunner et al., (2018), Dubin et al. (2021), Furue (2020), and Martin et al. (2020).
*IL31*	IL- 31		Itch, eczema severity	Akdis et al. (2020), Brunner et al., (2017), and Yang et al. (2020).
*IL33*	IL-33		Alarmin, promotes type 2 cytokine responses, asthma, and AD severity	Akdis et al. (2020), Salimi et al. (2013), and Yang et al. (2020).
*SPINK5*	Serine protease inhibitor, Kazal type 5		Skin barrier function, early-onset AD, disease severity, and food allergies in children with AD	Akdis et al. (2020), Dežman et al. (2017), Kusunoki et al. (2005), and Lan et al. (2011).
*STAT6*	Signal transducer and activator of transcription 6		Eczema, allergies	Furue (2020) and Tamura et al. (2001).
*TLR2*	Toll-like receptor 2		Asthma, skin infections, eczema severity	Martin et al. (2020), Niebuhr et al. (2009), and Yang et al. (2020).

Abbreviation: AD, atopic dermatitis; STAT6, signal transducer and activator of transcription 6; TLR2, toll-like receptor 2.

Other genes were analyzed by targeted sequencing of all protein-coding regions.

1 Gene analyzed by genotyping selected variants.

2 Gene partially analyzed by amplicon sequencing.

After quality control and variant annotation, a total of 247 variant loci were identified in the 140 patients with AD in the 13 genes studied by sequencing (Supplementary Table S1). Of the detected variants, 13 had a previous association with AD, asthma, allergy, or eosinophil counts with modest effect sizes, but their frequency in our cohort was similar to that in population cohorts (gnomAD database, version 2.1) (Table 5) (Karczewski et al., 2020).

**Table 5:** Variants Identified in Amplicon Sequencing with Previous Associations with Atopic Dermatitis, Eczema, Asthma, or Allergy

Gene	Chr	Position	Reference Allele	Alternative Allele	rsNumber	Frequency			Previous Associations		
						Study Cohort	gnomAD exome_ FIN	gnomAD genome_ FIN	GWAS Catalog	UK Biobank	Finngen F8
*IL33*	9	6253571	C	T	rs10975519	0.364	0.3515	0.3444	NS	Decreased eosinophil count and percentage, suggestive: protective for asthma	NS
*IL33*	9	6253710	G	C	rs10975520	0.364	NA	0.345	NA	Decreased eosinophil count and percentage, suggestive: protective for asthma	NS
*IL33*	9	6256292	G	A	rs1048274	0.361	NA	0.3447	NA	Decreased eosinophil count and percentage, suggestive: protective for asthma	NS
*IL4R*	16	27356203	A	G	rs1805010	0.364	0.3657	0.3673	NA	Increased risk for asthma, increased eosinophil count	Increased risk for asthma
*IL4R*	16	27356224	G	A	rs144651842	0.045-0.132 (low cover. region)	0.0866	0.0885	NA	NA	Protective for asthma and allergy
*IL4R*	16	27356359	C	T	rs2074572	0.277	0.2851	0.29	Increased eosinophil count	Increased risk for asthma, increased eosinophil count	NA
*IL4R*	16	27373980	C	T	rs1805013	0.046	0.028	0.0303	Suggestive association: earlier asthma age of onset	NS	NS
*IL4R*	16	27374400	A	G	rs1801275	0.200	0.1979	0.2011	Increased eosinophil count	Increased eosinophil count	NS
*STAT6*	12	57492996	G	A	rs841718	0.554	NA	0.5533	NA	Slightly increased risk for asthma	NS
*STAT6*	12	57493727	T	G	rs3024971	0.046	0.0408	0.0495	Protective for asthma, allergy eczema, lower eosinophil counts	Protective for asthma, allergy, decreased eosinophil count	NS
*IL22*	12	68646521	T	C	rs2227491	0.482	0.5474	0.5387	Increased risk of eczema	NS	Increased risk for atopic dermatitis
*IL13*	5	131995964	A	G	rs20541	0.625	0.6219	0.6095	Protective for eczema, asthma, allergy, and lower IgE. Increased risk for psoriasis	Protective for asthma, eczema/dermatitis, decreased eosinophil number	Protective for atopic dermatitis, asthma, and allergy
*LOC105379176*	5	132018169	C	A	rs2243290	0.343	0.3517	0.3607	NA	Increased risk for asthma and suggestive risk for eczema/dermatitis	Increased risk for atopic dermatitis and asthma

Abbreviations: Chr, chromosome; NA, not available; NS, not significant; STAT6, signal transducer and activator of transcription 6.

Variants more common in the study cohort although statistically not significant are bolded.

To identify putative high-impact variants, we filtered for rare, exonic variants causing likely LoF or amino acid change in the protein. A total of 116 of 247 variants were exonic, 69 were nonsynonymous, and 30 were rare or novel (frequency < 0.01 in gnomAD) (Figure 1d and Table 6 and Supplementary Table S2). LoF variants were not detected, but it should be noted that analyses for intronic deletions/duplications and copy number variation were not performed. Most of the rare variation was found in *DOCK8* (21 missense variants at 13 loci in 19 patients), biallelic LoF variants of which cause autosomal recessive hyper-IgE syndrome (Aydin et al., 2015). When we used a Combined Annotation Dependent Depletion score cutoff of 15—the median value for all possible canonical nonsynonymous and splice variants in Combined Annotation Dependent Depletion—nine *DOCK8* missense variants in 11 patients were considered potentially harmful (Kircher et al., 2014). This included two patients with two such *DOCK8* variants (NM_203447:c.A3535T p.S1179C/c.A4019G p.Y1340C and c.C305T p.T102I/c.C986T p.A329V), but it is not known whether these variants occurred in *cis* or *trans.* These two patients had parental AD, moderate disease, normal serum total IgE levels, slightly elevated eosinophil counts (0.40−0.53 E9/l), and positive aeroallergen sensitizations at baseline. They had no severe or frequent infections. One patient (IgE 277 kU/l) was diagnosed with epilepsy at 36 months. The other patient had high IgE and eosinophil levels (1,133 kU/l and 0.66 E9/l, respectively) as well as both food and aeroallergen sensitizations at the study end.

**Table 6:** Rare Variants Summary

Gene	Variant Loci	Variants	Patients	Information
*DOCK8*	13	21	19	Two patients with two variants (no knowledge of whether in *cis* or *trans*)
*IL4R*	4	9	8	One patient with two variants (no knowledge of whether in *cis* or *trans*)
*STAT6*	3	3	3	
*IL22*	1	1	1	
*SPINK5*	2	3	3	
*FLG*	4	4	4	
*TLR2*	2	2	2	
*CLDN1*	1	1	1	
*IL4*	0	0	0	
*IL13*	0	0	0	
*IL17A*	0	0	0	
*IL31*	0	0	0	
*IL33*	0	0	0	

Abbreviations: STAT6, signal transducer and activator of transcription 6; TLR2, toll-like receptor 2.

**Figure 3:**
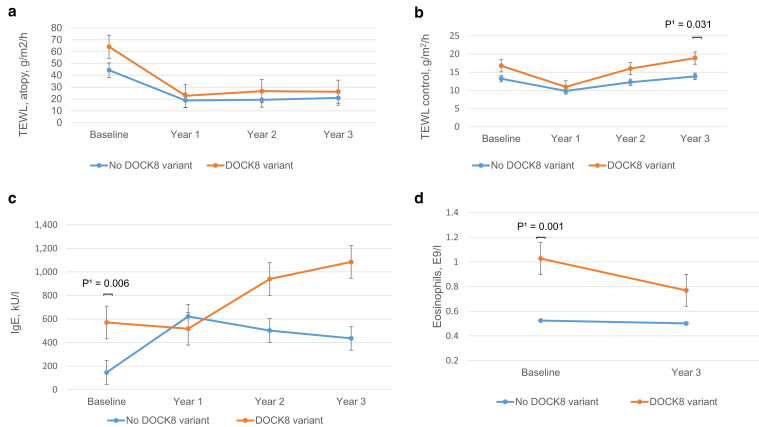
**Change in eczema treatment parameters (estimated marginal means) during the study in patients with and without possibly pathogenic *DOCK8* variant.** In this figure, *DOCK8* variant refers to rare *DOCK8* missense variants predicted to be potentially pathogenic. (**a**) TEWL (g/m2/h) at the eczema site. (**b**) TEWL (g/m2/h) at the control site. (**c**) IgE (kU/l). (**d**) Eosinphils (E9/l). The superscript number (^1^) denotes repeated measures ANOVA with Bonferroni correction. TEWL, transepidermal water loss.

Carriers of potentially harmful *DOCK8* variants (n = 10) had significantly increased total IgE and eosinophil counts in comparison with noncarriers (n = 110) both at baseline (IgE: 374 vs. 70 kU/l, *P* = 0.003; eosinophils: 0.71 vs. 0.44 E9/l, *P* = 0.025) and at 36 months (IgE: 671 vs. 147 kU/l, *P* = 0.002; eosinophils: 0.59 vs. 0.38 E9/l, *P* = 0.032). *FLG* LoF carriers were excluded from this analysis. Clinical data in relation to *DOCK8* status are shown in Supplementary Table S3, and change in eczema treatment parameters (estimated marginal means) during the study in patients with and without possibly pathogenic *DOCK8* variants is shown in Figure 3a−d.

## Discussion

Our study provides additional information on *FLG* LoF-variant carriers by presenting Finnish pediatric patients with moderate-to-severe AD. Many *FLG* variants have previously been shown to associate with AD (Brown et al., 2012; Luukkonen et al., 2017; Martin et al., 2020). However, these variants are only found in around 15−50% of patients with AD, and similarly, up to 40% of the carriers develop no AD at all (Palmer et al., 2006). In adult patients with AD, reduced *FLG* gene expression can occur in keratinocytes in the presence of IL-4 and IL-13 also without an *FLG* variant, whereas a similar finding was not made in a cohort of pediatric patients with early-onset AD (Esaki et al., 2016; Howell et al., 2009). Instead, barrier defects due to a reduced amount of epidermal lipids and tight junctions, such as claudins, have been seen in early pediatric AD (Bussmann et al., 2011).

In our pediatric cohort, *FLG* LoF variants were found in 14.3% (20 of 140) of the patients. The combined *FLG* LoF-variant frequency was significantly higher in study patients than in controls (7.14 % vs. 2.34%). This is slightly higher than the 5.6% *FLG* LoF-variant frequency previously reported for a Finnish adult AD cohort (Luukkonen et al., 2017). However, when the *FLG* LoF variants were tested individually, only the Arg2447Ter variant (n = 8) showed a statistically significant association with AD in our cohort. The detection of other associations was restricted by the small size of the cohort, which can be seen as a notable limitation in this study. The *FLG* LoF-variant carriers presented with higher TEWL values than noncarriers both at baseline (Ser761fs) and at previous eczema sites at 36 months (combined *FLG* LoF Arg501Ter, Ser761fs, Arg2447Ter, or Ser3247Ter) consistent with epidermal barrier impairment. This is in line with previous findings showing that *FLG* LoF variants lead to reduced amounts of natural moisturizing factor in the skin (Kezic et al., 2008).

Previous research has shown that the genetic background of AD is heterogeneous, and there is considerable variation in the results of genetic association studies done in cohorts of varying sizes and ethnicity. In addition, the frequency of specific *FLG* LoF variants in people of African or Asian ancestry differs from those of European ancestry (Wong et al., 2018; Zhu et al., 2021). For instance, *FLG* Pro478Ser and c.3321delA are prominent variants in Asia (Kim et al., 2019; On et al., 2017), whereas Arg501Ter and Ser3249Ter are the two most common variants in northern Europe (Brown et al., 2012; Sandilands et al., 2009). The association between *FLG* variants and AD is less clear in people of African ancestry (Nomura and Kabashima, 2021). In the Finnish adult patients with AD, carrier frequencies of Arg501Ter, Ser761fs, and Ser3247Ter were lower than their reported frequencies in other European populations, whereas frequencies of Arg2447Ter, Gln1754Ter, and Ser1020Ter were slightly higher in Finns (Luukkonen et al., 2017 and current study). Because amplicon sequencing failed to cover most of the *FLG* gene, other rare or, to our knowledge, previously unreported *FLG* LoF variants could not be detected in our study, and hence the reported total *FLG* LoF frequency in our cohort may be an underestimation.

*FLG* features intragenic copy number variation with allelic variation of either 10, 11, or 12 FLG repeats, which may affect the expressed FLG amount as well as urocanic acid concentration in the epidermis (Brown et al., 2012). In the Irish population, the rs12730241-A allele was used as a marker of the FLG allele with 12 repeats and was found to be protective against AD (Brown et al., 2012). Similarly, this variant was associated with a reduced risk of AD among European American subjects and in the Western Siberian population (Gao et al., 2009; Komova et al., 2014). However, in African Americans and in the previous Finnish adult AD work, rs12730241 showed an opposite effect, conferring instead increased risk of AD (Gao et al., 2009; Luukkonen et al., 2017). Moreover, Fernandez et al. (2017) found no association between AD severity and FLG repeat number in the Ethiopian population. In our Finnish pediatric AD cohort, we saw a modest association with AD for rs12730241 and another variant in linkage disequilibrium, rs6587667 (*FLG2*:Gly137Glu). The rs12730241-A allele was also associated with lower TEWL at previous eczema site at 36 months. These associations could be due to an effect of the FLG copy number, but it should be noted that a correlation between rs12730241 and the number of FLG repeats has not been confirmed in the Finnish population. Hence, it is possible that this association is driven by other genetic factors such as regulatory or structural variation present in the same haplotype. Currently, there are no validated markers for different numbers of FLG repeats in the Finnish population, and hence more in-depth analysis of the effect of *FLG* copy number variation in pediatric AD was out of scope for this study.

Although the significance of *FLG2* in AD remains unclear for the most part, an association between two *FLG2* variants and persistent AD was reported in African American patients (Margolis et al., 2014). Of these two variants, rs16833974/His1249Arg is extremely rare in the Finnish population (gnomAD FIN frequency = 0.0001592), but the rs12568784/Ser2377Ter variant is present at an allele frequency of 0.1309 and was thus included in our genotyping panel together with one other *FLG2* LoF variant (Thr1314fs) and four missense variants (Gly137Glu, Leu168Phe, Cys298Ser, and Cys298Arg). No association was seen between the rs12568784/Ser2377Ter variant and the risk of moderate-to-severe pediatric AD in Finns. However, our study did not compare nonpersistent with persistent patients with AD where the association for this variant was previously seen. Instead, we detected a modest association between the rs6587667 (*FLG2*:Gly137Glu) and risk of pediatric AD. However, owing to the linkage between this and the rs12730241 variant, the origin of this association signal needs further study.

In amplicon sequencing of 13 AD-related genes, we did not identify any LoF variants, previously reported pathogenic variants, or statistically significant enrichment of nonsynonymous coding region variants. However, we found it interesting that carriers of potentially harmful *DOCK8* variants had significantly increased total IgE and eosinophil counts in comparison with noncarriers both at baseline and at 36 months. All but one carrier had allergic sensitizations at the study end. The relation of *DOCK8* with AD has only been sparsely reported thus far, whereas DOCK8 deficiency due to recessive damaging variants is a well-documented cause of hyper IgE syndrome and a tendency to viral infections (Biggs et al., 2017; Boos et al., 2014; Jacob et al., 2019; Yamamura et al., 2017). Although AD is a complex multifactorial disease, the increase in IgE and eosinophil counts in carriers of potentially pathogenic *DOCK8* missense variants suggests that the role of *DOCK8* variation in AD should be further investigated in larger cohorts.

## Materials and Methods

### Patient cohort and eczema severity measures

Genetic analyses were carried out on 140 children who had moderate-to-severe AD and participated in a 3-year randomized open-label follow-up study between 2013 and 2019 (Figure 1a and Table 1, baseline demographics). AD severity was measured by Rajka−Langeland severity score, and clinical parameters included Eczema Area and Severity Index, Investigator’s Global Assessment score, eczema body surface area, and TEWL. TEWL was measured both at the control site (left forearm) and the eczema site at baseline. At 36 months, eczema TEWL was measured (Vapometer, Delfin Technologies, Kuopio, Finland) from the same site as the baseline. Treatment modalities were topical mild and moderate corticosteroids and tacrolimus. Response was defined as a decrease in eczema parameters.

### Sequencing and variant analysis

DNA was extracted from whole blood samples taken at 3 months using a salt precipitation−based method. Sequencing and genotyping were performed at the Sequencing Core of the Technology Centre of the Institute for Molecular Medicine Finland (Helsinki, Finland). Genotyping was performed using Sequenom MassARRAY system and iPLEX Gold assays (Agena Bioscience, San Diego, CA), as previously described by Luukkonen et al. (2017). Control samples (n = 1,664) obtained from the Health 2000 GenMets Study had previously been genotyped with the same method (Luukkonen et al., 2017). Quality control and analyses for genotype data were performed with PLINK (open-source genome analysis tool, version 1.90b5.3) (Chang et al., 2015). Analyses included samples with a maximum of two missing SNV calls and variants with missing call rates < 0.1.

Amplicon sequencing for *CLDN1*, *DOCK8*, *IL13*, *IL17A*, *IL22*, *IL31*, *IL33*, *IL4*, *IL4R*, *SPINK5*, signal transducer and activator of transcription 6 gene *STAT6*, toll-like receptor 2 gene *TLR2,* and *FLG* was performed using Illumina Truseq Custom Amplicon Kit and the MiSeq system (Illumina, San Diego, CA). Amplicon target information can be found in Supplementary Table S4. Reads were aligned to the GRCh37 human reference genome assembly utilizing Bowtie2 (Langmead and Salzberg, 2012), and variant calling was done using an in-house pipeline as previously described (Rajala et al., 2015). Variant Call Format files were trimmed and combined using BCFtools (Li, 2011). To analyze germline variants, only variants with alternative/reference read frequency ratio >0.2 were included in analyses. In addition, recurrent polymerase chain reaction/alignment errors were removed manually after visual inspection of data on Integrative Genomics Viewer (Robinson et al., 2017). Variants were defined as heterozygous when the alternative/reference read frequency ratio was between 0.2 and 0.8 and homozygous when the ratio was >0.8. Variant annotations were performed with ANNOVAR (Wang et al., 2010). Rare variants were defined as having a frequency <0.01 in gnomAD exomes and genomes of Finnish origin (Karczewski et al., 2020). Gene-wise rare variant frequencies in the pediatric AD cohort were also compared with rare variant frequencies in gnomAD to estimate the enrichment of rare variation in the selected candidate genes. Variant frequencies in the study cohort were calculated by dividing the number of identified alternative alleles by the number of samples with a minimum of 10× coverage at the site. Variant pathogenicity was estimated with Combined Annotation Dependent Depletion (Kircher et al., 2014) and REVEL (Ioannidis et al., 2016), and evolutionary conservation was evaluated with GERP++ (Cooper et al., 2010). Information for variants with previous associations to AD, eczema, allergy, IgE levels, or eosinophil numbers was sought from the following publicly available online databases: FinnGen F6 (FinnGen, 2022), UK Biobank (Canela-Xandri et al., 2018; GeneAtlas, 2017), and The NHGRI-EBI GWAS catalog (Buniello et al., 2019).

### Ethical considerations and permits

All parents or legal guardians provided written informed consent. The ethics committee of the Helsinki University Central Hospital and the Finnish Medicines Agency approved the study protocol (222/13/03/03/2012, EudraCT2012-002412-95).

### Statistics

Statistical analyses for clinical parameters were performed with the statistical software package SPSS 24 and 25 for Windows software (IBM, New York, NY). Association of *FLG* and *FLG2* variants with AD was tested using Fisher's exact test with Lancaster's mid-*P* adjustment and 95% confidence interval in PLINK. False discovery rate correction was applied to adjust *P*-values for multiple testing. A significance cutoff of *P* < 0.05 was used for all analyses. Quantitative clinical parameters in relation to genotype status were compared with the Mann−Whitney *U* test. Continuous variables are presented as medians with 25−75th percentiles (quartile 1−quartile 3)*.* Categorical variables are presented as counts and percentages. Repeated measures ANOVA (general linear models) was used to analyze eczema severity parameters over time.

### Data availability statement

*DOCK8* variant data have been submitted to the Global Variome shared LOVD and can be found at http://databases.lovd.nl/shared/references/DOI:10.1016/j.xjidi.2023.100203

## ORCIDs

Miia Perälä: http://orcid.org/0000-0003-3794-3515

Meri Kaustio: http://orcid.org/0000-0002-3547-8843

Alexander Salava: http://orcid.org/0000-0001-5471-5894

Eveliina Jakkula: http://orcid.org/0000-0001-9687-7787

Anna S. Pelkonen: http://orcid.org/0000-0002-1482-8947

Janna Saarela: http://orcid.org/0000-0002-0853-6219

Anita Remitz: http://orcid.org/0000-0001-7224-5662

Mika J. Mäkelä: http://orcid.org/0000-0002-2933-3111

## Conflict of Interest

JS has received a research grant and speaker's honoraria from Sanofi-Genzyme and is a founder and minority shareholder of VEIL.AI. The remaining authors state no conflict of interest.

## Supplementary Material

Supplementary Data1

Supplementary Data2

Supplementary Data3

Supplementary Data4

## References

[bib1] Akdis C.A., Arkwright P.D., Brüggen M.C., Busse W., Gadina M., Guttman-Yassky E. (2020). Type 2 immunity in the skin and lungs. Allergy.

[bib2] Alfares A., Al-Khenaizan S., Al Mutairi F. (2017). Peeling skin syndrome associated with novel variant in FLG2 gene. Am J Med Genet A.

[bib3] Amat F., Soria A., Tallon P., Bourgoin-Heck M., Lambert N., Deschildre A. (2018). New insights into the phenotypes of atopic dermatitis linked with allergies and asthma in children: an overview. Clin Exp Allergy.

[bib4] Asad S., Winge M.C., Wahlgren C.F., Bilcha K.D., Nordenskjöld M., Taylan F. (2016). The tight junction gene Claudin-1 is associated with atopic dermatitis among Ethiopians. J Eur Acad Dermatol Venereol.

[bib5] Aydin S.E., Kilic S.S., Aytekin C., Kumar A., Porras O., Kainulainen L. (2015). DOCK8 deficiency: clinical and immunological phenotype and treatment options - a review of 136 patients. J Clin Immunol.

[bib6] Benjamini Y., Hochberg Y. (1995). Controlling the false discovery rate: a practical and powerful approach to multiple testing. Journal of the Royal Statistical Society: Series B (Methodological).

[bib7] Bergmann S., von Buenau B., Vidal-Y-Sy S., Haftek M., Wladykowski E., Houdek P. (2020). Claudin-1 decrease impacts epidermal barrier function in atopic dermatitis lesions dose-dependently. Sci Rep.

[bib8] Bieber T., D'Erme A.M., Akdis C.A., Traidl-Hoffmann C., Lauener R., Schäppi G. (2017). Clinical phenotypes and endophenotypes of atopic dermatitis: where are we, and where should we go?. J Allergy Clin Immunol.

[bib9] Biggs C.M., Keles S., Chatila T.A. (2017). DOCK8 deficiency: insights into pathophysiology, clinical features and management. Clin Immunol.

[bib10] Bolling M.C., Jan S.Z., Pasmooij A.M.G., Lemmink H.H., Franke L.H., Yenamandra V.K. (2018). Generalized ichthyotic peeling skin syndrome due to FLG2 mutations. J Invest Dermatol.

[bib11] Boos A.C., Hagl B., Schlesinger A., Halm B.E., Ballenberger N., Pinarci M. (2014). Atopic dermatitis, STAT3- and DOCK8-hyper-IgE syndromes differ in IgE-based sensitization pattern. Allergy.

[bib12] Brown S.J., Kroboth K., Sandilands A., Campbell L.E., Pohler E., Kezic S. (2012). Intragenic copy number variation within filaggrin contributes to the risk of atopic dermatitis with a dose-dependent effect. J Invest Dermatol.

[bib71] Brunner P.M., Guttman-Yassky E., Leung D.Y. (2017). The immunology of atopic dermatitis and its reversibility with broad-spectrum and targeted therapies. J Allergy Clin Immunol.

[bib70] Brunner P.M., Israel A., Zhang N., Leonard A., Wen H.C., Huynh T. (2018). Early-onset pediatric atopic dermatitis is characterized by T_H_2/T_H_17/T_H_22-centered inflammation and lipid alterations. J Allergy Clin Immunol.

[bib13] Buniello A., MacArthur J.A.L., Cerezo M., Harris L.W., Hayhurst J., Malangone C. (2019). The NHGRI-EBI GWAS Catalog of published genome-wide association studies, targeted arrays and summary statistics 2019. Nucleic Acids Res.

[bib14] Bussmann C., Weidinger S., Novak N. (2011). Genetics of atopic dermatitis. J Dtsch Dermatol Ges.

[bib15] Canela-Xandri O., Rawlik K., Tenesa A. (2018). An atlas of genetic associations in UK Biobank. Nat Genet.

[bib16] Chan A., Terry W., Zhang H., Karmaus W., Ewart S., Holloway J.W. (2018). Filaggrin mutations increase allergic airway disease in childhood and adolescence through interactions with eczema and aeroallergen sensitization. Clin Exp Allergy.

[bib17] Chang C.C., Chow C.C., Tellier L.C.A.M., Vattikuti S., Purcell S.M., Lee J.J. (2015). Second-generation PLINK: rising to the challenge of larger and richer datasets. GigaScience.

[bib18] Cooper G.M., Goode D.L., Ng S.B., Sidow A., Bamshad M.J., Shendure J. (2010). Single-nucleotide evolutionary constraint scores highlight disease-causing mutations. Nat Methods.

[bib19] De Benedetto A., Rafaels N.M., McGirt L.Y., Ivanov A.I., Georas S.N., Cheadle C. (2011). Tight junction defects in patients with atopic dermatitis. J Allergy Clin Immunol.

[bib20] Dežman K., Korošec P., Rupnik H., Rijavec M. (2017). SPINK5 is associated with early-onset and CHI3L1 with late-onset atopic dermatitis. Int J Immunogenet.

[bib21] Drislane C., Irvine A.D. (2020). The role of filaggrin in atopic dermatitis and allergic disease. Ann Allergy Asthma Immunol.

[bib22] Dubin C., Del Duca E., Guttman-Yassky E. (2021). The IL-4, IL-13 and IL-31 pathways in atopic dermatitis. Expert Rev Clin Immunol.

[bib24] Engelhardt K.R., Gertz M.E., Keles S., Schäffer A.A., Sigmund E.C., Glocker C. (2015). The extended clinical phenotype of 64 patients with dedicator of cytokinesis 8 deficiency. J Allergy Clin Immunol.

[bib25] Esaki H., Brunner P.M., Renert-Yuval Y., Czarnowicki T., Huynh T., Tran G. (2016). Early-onset pediatric atopic dermatitis is TH2 but also TH17 polarized in skin. J Allergy Clin Immunol.

[bib26] Fernandez K., Asad S., Taylan F., Wahlgren C.F., Bilcha K.D., Nordenskjöld M. (2017). Intragenic copy number variation in the filaggrin gene in Ethiopian patients with atopic dermatitis. Pediatr Dermatol.

[bib27] FinnGen. FinnGen F6 Release 01/24/2022, https://r6.finngen.fi/; (accessed 9 March 2022).

[bib28] Furue M. (2020). Regulation of filaggrin, loricrin, and involucrin by IL-4, IL-13, IL-17A, IL-22, AHR, and NRF2: pathogenic implications in atopic dermatitis. Int J Mol Sci.

[bib29] Gao P.S., Rafaels N.M., Hand T., Murray T., Boguniewicz M., Hata T. (2009). Filaggrin mutations that confer risk of atopic dermatitis confer greater risk for eczema herpeticum. J Allergy Clin Immunol.

[bib30] GeneAtlas, GeneAtlas, http://geneatlas.roslin.ed.ac.uk/; 2017. (accessed 9 March 2022).

[bib31] Howell M.D., Kim B.E., Gao P., Grant A.V., Boguniewicz M., Debenedetto A. (2009). Cytokine modulation of atopic dermatitis filaggrin skin expression. J Allergy Clin Immunol.

[bib32] Ioannidis N.M., Rothstein J.H., Pejaver V., Middha S., McDonnell S.K., Baheti S. (2016). REVEL: an ensemble method for predicting the pathogenicity of rare missense variants. Am J Hum Genet.

[bib33] Jacob M., Bin Khalaf D., Alhissi S., Arnout R., Alsaud B., Al-Mousa H. (2019). Quantitative profiling of cytokines and chemokines in DOCK8-deficient and atopic dermatitis patients. Allergy.

[bib34] Karczewski K.J., Francioli L.C., Tiao G., Cummings B.B., Alföldi J., Wang Q. (2020). The mutational constraint spectrum quantified from variation in 141,456 humans. Nature.

[bib35] Kezic S., Kemperman P.M., Koster E.S., de Jongh C.M., Thio H.B., Campbell L.E. (2008). Loss-of-function mutations in the filaggrin gene lead to reduced level of natural moisturizing factor in the stratum corneum. J Invest Dermatol.

[bib36] Kim B.J., Wang H.Y., Lee H., Lee S.Y., Hong S.J., Choi E.H. (2019). Clinical characteristics and genetic variations in early-onset atopic dermatitis patients. Ann Dermatol.

[bib37] Kircher M., Witten D.M., Jain P., O'Roak B.J., Cooper G.M., Shendure J. (2014). A general framework for estimating the relative pathogenicity of human genetic variants. Nat Genet.

[bib38] Komova E.G., Shintyapina A.B., Makarova S.I., Ivanov M.K., Chekryga E.A., Kaznacheeva L.F. (2014). Filaggrin mutations in a Western siberian population and their association with atopic dermatitis in children. Genet Test Mol Biomarkers.

[bib39] Kusunoki T., Okafuji I., Yoshioka T., Saito M., Nishikomori R., Heike T. (2005). SPINK5 polymorphism is associated with disease severity and food allergy in children with atopic dermatitis. J Allergy Clin Immunol.

[bib40] Lan C.C., Tu H.P., Wu C.S., Ko Y.C., Yu H.S., Lu Y.W. (2011). Distinct SPINK5 and IL-31 polymorphisms are associated with atopic eczema and non-atopic hand dermatitis in Taiwanese nursing population. Exp Dermatol.

[bib41] Langmead B., Salzberg S.L. (2012). Fast gapped-read alignment with Bowtie 2. Nat Methods.

[bib42] Li H. (2011). A statistical framework for SNP calling, mutation discovery, association mapping and population genetical parameter estimation from sequencing data. Bioinformatics.

[bib43] Liang Y., Chang C., Lu Q. (2016). The genetics and epigenetics of atopic dermatitis-filaggrin and other polymorphisms. Clin Rev Allergy Immunol.

[bib44] Løset M., Brown S.J., Saunes M., Hveem K. (2019). Genetics of atopic dermatitis: from DNA sequence to clinical relevance. Dermatology.

[bib45] Luukkonen T.M., Kiiski V., Ahola M., Mandelin J., Virtanen H., Pöyhönen M. (2017). The value of FLG null mutations in predicting treatment response in atopic dermatitis: an observational study in Finnish patients. Acta Derm Venereol.

[bib46] Makino T., Mizawa M., Yamakoshi T., Takaishi M., Shimizu T. (2014). Expression of filaggrin-2 protein in the epidermis of human skin diseases: a comparative analysis with filaggrin. Biochem Biophys Res Commun.

[bib47] Margolis D.J., Gupta J., Apter A.J., Ganguly T., Hoffstad O., Papadopoulos M. (2014). Filaggrin-2 variation is associated with more persistent atopic dermatitis in African American subjects. J Allergy Clin Immunol.

[bib48] Martin M.J., Estravís M., García-Sánchez A., Dávila I., Isidoro-García M., Sanz C. (2020). Genetics and epigenetics of atopic dermatitis: an updated systematic review. Genes (Basel).

[bib49] Mohamad J., Sarig O., Godsel L.M., Peled A., Malchin N., Bochner R. (2018). Filaggrin 2 deficiency results in abnormal cell-cell adhesion in the cornified cell layers and causes peeling skin syndrome type A. J Invest Dermatol.

[bib50] Niebuhr M., Lutat C., Sigel S., Werfel T. (2009). Impaired TLR-2 expression and TLR-2-mediated cytokine secretion in macrophages from patients with atopic dermatitis. Allergy.

[bib51] Nomura T., Kabashima K. (2021). Advances in atopic dermatitis in 2019–2020: endotypes from skin barrier, ethnicity, properties of antigen, cytokine profiles, microbiome, and engagement of immune cells. J Allergy Clin Immunol.

[bib52] On H.R., Lee S.E., Kim S.E., Hong W.J., Kim H.J., Nomura T. (2017). Filaggrin mutation in Korean patients with atopic dermatitis. Yonsei Med J.

[bib53] Palmer C.N., Irvine A.D., Terron-Kwiatkowski A., Zhao Y., Liao H., Lee S.P. (2006). Common loss-of-function variants of the epidermal barrier protein filaggrin are a major predisposing factor for atopic dermatitis. Nat Genet.

[bib54] Pendaries V., Le Lamer M., Cau L., Hansmann B., Malaisse J., Kezic S. (2015). In a three-dimensional reconstructed human epidermis filaggrin-2 is essential for proper cornification. Cell Death Dis.

[bib57] Rajala H.L.M., Olson T., Clemente M.J., Lagström S., Ellonen P., Lundan T. (2015). The analysis of clonal diversity and therapy responses using STAT3 mutations as a molecular marker in large granular lymphocytic leukemia. Haematologica.

[bib58] Robinson J.T., Thorvaldsdóttir H., Wenger A.M., Zehir A., Mesirov J.P. (2017). Variant review with the integrative genomics viewer. Cancer Res.

[bib59] Salimi M., Barlow J.L., Saunders S.P., Xue L., Gutowska-Owsiak D., Wang X. (2013). A role for IL-25 and IL-33-driven type-2 innate lymphoid cells in atopic dermatitis. J Exp Med.

[bib60] Sandilands A., Sutherland C., Irvine A.D., McLean W.H. (2009). Filaggrin in the frontline: role in skin barrier function and disease. J Cell Sci.

[bib61] Smith F.J., Irvine A.D., Terron-Kwiatkowski A., Sandilands A., Campbell L.E., Zhao Y. (2006). Loss-of-function mutations in the gene encoding filaggrin cause ichthyosis vulgaris. Nat Genet.

[bib62] Tamura K., Arakawa H., Suzuki M., Kobayashi Y., Mochizuki H., Kato M. (2001). Novel dinucleotide repeat polymorphism in the first exon of the STAT-6 gene is associated with allergic diseases. Clin Exp Allergy.

[bib63] Wang K., Li M., Hakonarson H. (2010). ANNOVAR: functional annotation of genetic variants from high-throughput sequencing data. Nucleic Acids Res.

[bib64] Wong X.F.C.C., Denil S.L.I.J., Foo J.N., Chen H., Tay A.S.L., Haines R.L. (2018). Array-based sequencing of filaggrin gene for comprehensive detection of disease-associated variants. J Allergy Clin Immunol.

[bib65] Wu Z., Hansmann B., Meyer-Hoffert U., Gläser R., Schröder J.M. (2009). Molecular identification and expression analysis of filaggrin-2, a member of the S100 fused-type protein family. PLoS One.

[bib66] Yamamura K., Uruno T., Shiraishi A., Tanaka Y., Ushijima M., Nakahara T. (2017). The transcription factor EPAS1 links DOCK8 deficiency to atopic skin inflammation via IL-31 induction. Nat Commun.

[bib67] Yang G., Seok J.K., Kang H.C., Cho Y.Y., Lee H.S., Lee J.Y. (2020). Skin barrier abnormalities and immune dysfunction in atopic dermatitis. Int J Mol Sci.

[bib68] Zhu Y., Mitra N., Feng Y., Tishkoff S., Hoffstad O., Margolis D. (2021). FLG variation differs between European Americans and African Americans. J Invest Dermatol.

[bib55] Perälä M., Salava A., Malmberg P, Pelkonen A.S., Mäkelä M.J., Remitz A. Topical tacrolimus and corticosteroids in childhood moderate-to-severe atopic dermatitis with impact on airways: A long-term randomized open-label study. Clin Exp Dermatol. 2023. 10.1093/ced/llad098. Epub ahead of print. PMID: 36916653. 36916653

